# Metabolomics approaches in pancreatic adenocarcinoma: tumor metabolism profiling predicts clinical outcome of patients

**DOI:** 10.1186/s12916-017-0810-z

**Published:** 2017-03-16

**Authors:** S. Battini, F. Faitot, A. Imperiale, A. E. Cicek, C. Heimburger, G. Averous, P. Bachellier, I. J. Namer

**Affiliations:** 1 ICube, UMR 7357 University of Strasbourg/CNRS, Strasbourg, France; 2 Department of Visceral Surgery and Transplantation, Hautepierre Hospital, University Hospitals of Strasbourg, Strasbourg, France; 3 FMTS, Faculty of Medicine, Strasbourg, France; 4 Department of Biophysics and Nuclear Medicine, Hautepierre Hospital, University Hospitals of Strasbourg, 1, Avenue Molière, Strasbourg, Cedex 67098 France; 5 Computational Biology Department, Carnegie Mellon University, Pittsburgh, PA USA; 6 Computer Engineering Department, Bilkent University, Ankara, Turkey; 7 Department of Pathology, Hautepierre Hospital, University Hospitals of Strasbourg, Strasbourg, France

**Keywords:** Metabolomics, HRMAS, NMR, Biomarker, Pancreatic adenocarcinoma, Long-term survival

## Abstract

**Background:**

Pancreatic adenocarcinomas (PAs) have very poor prognoses even when surgery is possible. Currently, there are no tissular biomarkers to predict long-term survival in patients with PA. The aims of this study were to (1) describe the metabolome of pancreatic parenchyma (PP) and PA, (2) determine the impact of neoadjuvant chemotherapy on PP and PA, and (3) find tissue metabolic biomarkers associated with long-term survivors, using metabolomics analysis.

**Methods:**

^1^H high-resolution magic angle spinning (HRMAS) nuclear magnetic resonance (NMR) spectroscopy using intact tissues was applied to analyze metabolites in PP tissue samples (*n* = 17) and intact tumor samples (*n* = 106), obtained from 106 patients undergoing surgical resection for PA.

**Results:**

An orthogonal partial least square-discriminant analysis (OPLS-DA) showed a clear distinction between PP and PA. Higher concentrations of myo-inositol and glycerol were shown in PP, whereas higher levels of glucose, ascorbate, ethanolamine, lactate, and taurine were revealed in PA. Among those metabolites, one of them was particularly obvious in the distinction between long-term and short-term survivors. A high ethanolamine level was associated with worse survival. The impact of neoadjuvant chemotherapy was higher on PA than on PP.

**Conclusions:**

This study shows that HRMAS NMR spectroscopy using intact tissue provides important and solid information in the characterization of PA. Metabolomics profiling can also predict long-term survival: the assessment of ethanolamine concentration can be clinically relevant as a single metabolic biomarker. This information can be obtained in 20 min, during surgery, to distinguish long-term from short-term survival.

**Electronic supplementary material:**

The online version of this article (doi:10.1186/s12916-017-0810-z) contains supplementary material, which is available to authorized users.

## Background

Pancreatic adenocarcinomas (PAs) are extremely aggressive cancers and have one of the poorest prognoses among all cancers [1]. With an estimated 48,960 new cases in 2015 in the USA, pancreatic cancer is the twelfth most common cancer, representing 3.0% of all the new cancers diagnosed in the USA [2]. The majority of pancreatic cancers are pancreatic ductal adenocarcinomas and are localized in the head of the pancreas [3, 4].

Surgery is the only potentially curative treatment for PA. Pancreatic surgery is associated with significant morbidity and mortality. Indeed the mortality rate, even in highly specialized centers, ranges from 2% to 5%, and morbidity can be as high as 70%, especially in left pancreatic resection [5, 6]. The extension of lymphadenectomy, with its high morbidity and low evidence-based data, is actually a matter for debate [7, 8]. In recent years, a significant shift towards targeted surgical interventions has been proposed, relying on accurate characterization of PA, made possible by preoperative imaging to help evaluate its resectability [9].

Even though computed tomography (CT) and magnetic resonance imaging (MRI) studies now precisely differentiate resectable or locally advanced versus unresectable PA, there is currently a debate on whether survival can be predicted in resected patients on the basis of imaging. Notably, the vascular contacts may be complex to assess, especially after endoprosthesis or after radio- and/or chemotherapy, where the prognostic value of imaging is still debated [10]. The main prognostic factors for survival after cephalic duodeno-pancreatectomy (CDP) are histological parameters, namely R0 margins, nodal status, and differentiation [11, 12]. Although technical breakthroughs have been achieved in the field of pancreatic surgery, it has nevertheless been shown that the rate of R0 resection is rarely more than 20% and that it consistently impacts survival [13, 14]. Nodal extension depends on the extension of lymphadenectomy, as shown by the impact of invaded node-to-total examined node ratio [15]. However, the benefit of extended lymphadenectomy in PA has not been demonstrated. Therefore, there is a lack of accurate prognostic factors, and currently no tissue biomarkers have been identified to predict long-term survival in patients with pancreatic cancer.

Gross examination and intraoperative extemporaneous microscopic examination are reliable for diagnosis, but cannot predict overall survival.

In recent years, metabolomics, or global metabolite profiling, has been used to investigate the metabolite changes associated with pancreatic cancers [16–21]. Metabolomics is the latest stage of the multi-omics approaches. After genomics, transcriptomics, and proteomics, metabolomics has been generating increasing interest in scientific and medical communities in the past few years, particularly in oncology [22] and more precisely in pancreatic cancers. Currently, well-recognized tools for metabolomics are nuclear magnetic resonance (NMR) spectroscopy and gas (GC-MS) or liquid chromatography-mass spectrometry (LC-MS). ^1^H high-resolution magic angle spinning (HRMAS) NMR spectroscopy technology is particularly suitable for the analysis of small samples of intact tissue. This technique avoids the need for chemical extraction procedures or for handling the samples, both of which are required by MS and liquid-state NMR. HRMAS NMR spectroscopy enables identification and quantification of several metabolites from spectra with excellent resolution and signal-to-noise ratio.

Beyond serum markers [16, 19], better characterization of pancreatic tissue would be of particular interest in PA. Consequently, there is a need for accurate tissue biomarkers that could help surgeons distinguish between long-term and short-term survivors. The aims of this study were thus to (1) define the metabolome of pancreatic parenchyma (PP, healthy tissue) and PA, (2) determine the impact of neoadjuvant chemotherapy on healthy tissue (PP) and PA, and (3) by using metabolomics analysis, find metabolic biomarkers associated with long-term survival in patients with PA.

## Methods

### Patient population

This study included 123 samples obtained from 106 patients retrospectively selected after they had undergone PA resection, from May 2000 to March 2011, in the Department of Visceral Surgery and Transplantation (University Hospitals of Strasbourg, Hautepierre Hospital, Strasbourg, France). These patients fulfilled the following criteria: (1) histological diagnosis of PA, (2) all follow-up patients, (3) only patients with tumor-related deaths, (4) homogeneous adjuvant treatment using the same chemotherapy (gemcitabine) for all patients (and no radiotherapy), and (5) samples of pancreatic tissue collected just after resection and then snap-frozen in liquid nitrogen before storage.

Among the 106 samples obtained from patients with PA, there were:

- 44 samples from patients who did not receive any neoadjuvant chemotherapy
- 62 samples from patients who did

Among the 17 samples obtained from PP (healthy tissue), there were:

- 9 samples from patients who did not receive any neoadjuvant chemotherapy
- 8 samples from patients who did

Finally, two groups with extremely different prognoses (PA samples) were compared:

- Long-term survivors (survival >3 years), 8 samples, no neoadjuvant chemotherapy
- Short-term survivors (survival <1 year), 9 samples, no neoadjuvant chemotherapy

For this investigation, the tissue samples were obtained from the tumor bio-bank of Strasbourg University Hospitals. A written informed consent was given by all the included patients.

### Tissue sample preparation for HRMAS NMR spectroscopy

All tissue specimens were collected during surgery just after tumor removal and were snap-frozen in liquid nitrogen for –80 °C temperature storage. Then, the sample preparation was performed at a temperature of –20 °C. The amount of tissue used for the HRMAS analysis ranged from 15 mg to 20 mg. Each tissue sample was placed in a 30-μL disposable insert. Next, 8 μL of deuterium oxide with 0.75 weight percent 2,2,3,3-D4-3-(trimethylsilyl) propionic acid was added in every biopsy’s insert in order to get a chemical shift reference for the NMR spectrometer. Finally, inserts were kept at –80 °C until the HRMAS analysis was performed. The insert was placed in a 4-mm ZrO_2_ rotor just before the HRMAS analysis.

### HRMAS NMR data acquisition

All HRMAS NMR spectra were obtained on a Bruker Avance III 500 spectrometer (installed at Hautepierre Hospital, Strasbourg) operating at a proton frequency of 500.13 MHz and equipped with a 4 mm triple resonance gradient HRMAS probe (^1^H, ^13^C, and ^31^P). The temperature was maintained at 277.15 K throughout the acquisition time in order to reduce the effects of tissue degradation during the spectra acquisition. A one-dimensional (1D) proton spectrum using a Carr-Purcell-Meiboom-Gill (CPMG) pulse sequence was acquired with an interpulse delay of 285 μs and an acquisition time of 10 min for each tissue sample (Bruker GmbH, Germany). The number of loops was set to 328, giving the CPMG pulse train a total length of 93 ms. The chemical shift was calibrated to the peak of the methyl proton of l-lactate at 1.33 ppm. In order to confirm resonance assignments in a few representative samples, two-dimensional (2D) heteronuclear experiments (^1^H – ^13^C) were also recorded immediately after ending the 1D spectra acquisition. Metabolites were assigned using standard metabolite chemical shift tables available in the literature (Table 1) [23].

**Table 1:** NMR resonance assignments of the metabolites identified in samples of pancreatic intact tissues

	Metabolite	Group	^1^H chemical shift (ppm)	^13^C chemical shift (ppm)
1	Leucine	δCH_3_	0.95	23.43
		δ'CH_3_	0.95	24.75
2	Lactate	CH_3_	1.33	22.69
		**CH**	4.13	71.22
3	Glycine	**CH**_**2**_	3.56	44.05
4	Glycerol	CH_2_-OH (d 2X)	3.55	65.03
		**CH**_**2**_ **-OH (u 2X)**	3.64	65.07
		CH-OH	3.77	74.69
5	Glutamine	αCH	3.77	57.23
		**γCH**_**2**_	2.44	33.52
6	Serine	γCH_2_	3.97	62.90
7	Taurine	CH_2_-NH_3_+	3.27	50.01
		**CH**_**2**_ **-SO**_**3**_^**-**^	3.43	37.93
8	Valine	γ'CH_3_	1.04	20.44
		αCH-NH_2_	3.60	63.05
9	Arginine	γCH_2_	1.65	25.90
		βCH_2_	1.92	30.13
		δCH_2_	3.22	43.23
10	β-Glucose	C6H(d)	3.89	63.48
		**C1H**	4.65	98.70
11	α-Glucose	CH_2_	3.83	62.08
		**C1H**	5.22	94.94
12	Lysine	δCH_2_	1.73	29.17
		βCH_2_	1.90	32.48
		γCH_2_	1.91	30.25
13	Glutamic acid	βCH_2_	2.08	29.67
		**γCH**_**2**_	2.35	35.96
14	Alanine	βCH_3_	1.48	18.87
15	Myo-Inositol	(CH)2	3.54	73.81
		(CH)2	3.63	75.11
		CH	4.05	74.79
16	Ornithine	αCH-NH_2_	3.77	57.05
		δCH_2_-NH_2_	3.05	41.83
		β-CH_2_	1.93	30.28
		γ-CH_2_	1.74	25.52
17	3-Hydroxybutyric acid	CH_3_	1.20	24.30
18	Creatine	**CH**_**2**_	3.93	56.23
19	Choline	**N** ^**+**^ **-(CH**_**3**_ **)**_**3**_	3.23	56.48
20	Fatty acids (a)	(1)CH_2_	1.30	32.16
21	Fatty acids (b)	CH_2_	2.80	28.50
22	Fatty acids (c)	(1)CH_2_	2.26	36.60
23	Succinic acid	**(CH**_**2**_ **)2**	2.39	34.00
24	Ascorbate	**CH-O**	4.52	80.87
25	Ethanolamine	**CH**_**2**_ **-NH**_**2**_	3.13	43.90
26	Phosphorylcholine	**CH**_**2**_ **-O**	4.11	63.60
		N^+^-(CH_3_)_3_	3.22	56.57
27	Threonine	**βCH**	4.25	68.50
28	Glycerophosphocholine	N^+^-(CH_3_)_3_	3.21	56.56
		βCH_2_	3.72	68.49
		**αCH**_**2**_	4.33	62.16
		CH_2_OH	3.93	73.32
		CH_2_-HPO_4_(d)	3.89	69.22
29	Tyrosine	**meta CH** ortho CH βCH_2_(d)	6.88 7.18 3.02	118.44 133.30 39.40
30	Phenylalanine	ortho CH **para CH** meta CH	7.31 7.36 7.42	131.91 132.28 131.59
31	Aspartic acid	**βCH**_**2**_ **(d)** βCH_2_(u)	2.63 2.81	40.20 40.82

Each peak in the 2D spectra represents a correlation ^1^H – ^13^C. Metabolites were assigned using standard metabolite chemical shift tables available in the literature [23]. The groups in bold text were used to perform the metabolites’ quantification

### HRMAS NMR data processing and statistical analyses

The HRMAS NMR data processing and the metabolites’ quantification have been previously detailed [24]. Briefly, the region between 7.50 and 0.70 ppm of each 1D HRMAS NMR spectrum was automatically bucketed into integral regions of 0.01 ppm, using AMIX 3.9.14 software (Bruker GmbH, Germany). Once the data set was obtained, it was then exported and analyzed into SIMCA P (version 13.0.3, Umetrics AB, Umeå, Sweden). An orthogonal partial least square-discriminant analysis (OPLS-DA) was performed to analyze the data. The following OPLS-DA model was considered: PP versus PA (both without neoadjuvant chemotherapy). Two measurements of model quality were reported for OPLS-DA: R^ 2^Y and Q^2^. R^2^Y > 0.7 and Q^2^ >0.5 can be considered as a good predictor.

When the population is small, instead of applying OPLS-DA analysis, network analyses using the “algorithm to determine expected metabolite level alterations” using mutual information (ADEMA) are justified [25]. This is why ADEMA has been applied to metabolite quantification values. ADEMA evaluates the changes in groups of metabolites between the case and the control instead of analyzing metabolites one by one. ADEMA includes the metabolic network topology and uses mutual information to find out if those metabolites are biomarkers when considered together, and it can predict the expected change in direction per metabolite when the metabolic network topology is considered. The network was constructed using the Kyoto Encyclopedia of Genes and Genomes [26, 27] and Selway’s work [28].

The following groups of metabolites were compared related to involved metabolic pathways:

- Choline, phosphorylcholine, glycerophosphocholine, ethanolamine
- Glycerol, glucose
- Glucose, lactate
- Aspartate, threonine
- Glucose, glycine
- Aspartate, taurine
- Aspartate, succinate
- Glucose, ascorbate, glycine, glutamate
- Glutamate, glutamine
- Glutamate, glutamine, glycine
- Glutamate, glycine, creatine
- Tyrosine, phenylalanine

The metabolites were quantified using the PULCON method, which is a very accurate quantification method (with a very limited percentage of error [29]).

The repetition time used for this study (2 s) and the total acquisition time (10 min) are hence a good compromise in order to quantify the metabolites by HRMAS NMR spectroscopy of intact tissue. However, under these conditions, we cannot see the whole of the metabolites, but only the freer parts of them. Furthermore, the latter are underestimated about 20% in comparison with studies performing tissue extractions (data not shown). This method is widely used in the literature.

Metabolite quantification was performed using an external reference standard of lactate (3 μmol), scanned under the same analytical conditions as the tissue samples. Spectra were normalized according to sample weight. Peaks of interest were automatically defined by an in-house program using MATLAB 7.0 (MathWorks, Natick, MA, USA). Peak integration was then compared to the one obtained with the lactate reference and was corrected according to the number of protons. Only well-defined peaks with no overlapping in the 1D CPMG spectra were selected for quantification (Tables 2, 3, 4, and 5). Quantification results were expressed as nanomoles per milligram of tissue.

**Table 2:** Comparison between PP and PA (both without neoadjuvant chemotherapy)

Metabolite	No neoadjuvant chemotherapy	Mean (nmol/mg)	*p* value
Ascorbate	PP	0.3488 ± 0.2191	0.4454
	PA	0.3988 ± 0.2221	
Aspartic acid	PP	0.7577 ± 0.3284	0.2926
	PA	0.6229 ± 0.2255	
Choline	PP	1.5580 ± 0.5921	**0.0014**
	PA	0.8849 ± 0.3887	
Creatine	PP	1.7371 ± 0.9568	0.5047
	PA	1.3561 ± 0.5057	
Ethanolamine	PP	0.9314 ± 0.4502	**0.0226**
	PA	0.6188 ± 0.2620	
Glutamate	PP	2.9830 ± 0.8698	0.1114
	PA	2.4650 ± 0.7699	
Glycerol	PP	8.1840 ± 7.3988	**0.0037**
	PA	2.2750 ± 1.1994	
Glycine	PP	6.0920 ± 5.0908	**0.0005**
	PA	2.2663 ± 0.8577	
Glycerophosphocholine	PP	2.4215 ± 2.6084	0.3516
	PA	1.0319 ± 0.5029	
Lactate	PP	11.0040 ± 2.4733	**0.0006**
	PA	16.1370 ± 4.7272	
Phosphorylcholine	PP	1.1696 ± 0.6396	0.5201
	PA	0.9764 ± 0.5008	
Taurine	PP	2.9660 ± 1.9594	**0.0021**
	PA	4.4630 ± 1.4063	
Threonine	PP	1.1202 ± 0.2821	0.6512
	PA	1.1931 ± 0.4230	
Glutamine	PP	0.6855 ± 0.2518	0.6859
	PA	0.6394 ± 0.2272	
Succinic acid	PP	0.3068 ± 0.2121	0.1786
	PA	0.1762 ± 0.0763	
Glucose	PP	1.2247 ± 0.6158	0.7937
	PA	1.3141 ± 1.1020	
Tyrosine	PP	0.0521 ± 0.0443	0.0583
	PA	0.0741 ± 0.0472	
Phenylalanine	PP	0.1449 ± 0.0771	0.0866
	PA	0.0741 ± 0.0811	

Results of the Mann-Whitney *U* test. Metabolite differences between PP and PA, both without neoadjuvant chemotherapy (univariate analysis, nonparametric test)

*p* <0.05 are in boldface

**Table 3:** Impact of neoadjuvant chemotherapy on healthy tissue (PP)

Metabolite	Neoadjuvant chemotherapy	Mean (nmol/mg)	*p* value
Ascorbate	No	0.3488 ± 0.2191	0.6730
	Yes	0.3592 ± 0.1671	
Aspartic acid	No	0.7577 ± 0.3284	0.9626
	Yes	0.6730 ± 0.1776	
Choline	No	1.5580 ± 0.5921	0.7430
	Yes	1.7649 ± 0.7624	
Creatine	No	1.7371 ± 0.9568	0.5414
	Yes	1.7760 ± 0.6579	
Ethanolamine	No	0.9314 ± 0.4502	0.6058
	Yes	1.1115 ± 0.5408	
Glutamate	No	2.9830 ± 0.8698	0.8148
	Yes	2.3800 ± 0.4264	
Glycerol	No	8.1840 ± 7.3988	0.6730
	Yes	8.2990 ± 7.2904	
Glycine	No	6.0920 ± 5.0908	0.5414
	Yes	6.5590 ± 5.7401	
Glycerophosphocholine	No	2.4215 ± 2.6084	0.9626
	Yes	2.2208 ± 2.1689	
Lactate	No	11.0040 ± 2.4733	0.3704
	Yes	9.6770 ± 2.1588	
Phosphorylcholine	No	1.1690 ± 0.6396	0.8148
	Yes	0.9469 ± 0.3644	
Taurine	No	2.9660 ± 1.9594	0.9626
	Yes	2.7060 ± 1.3103	
Threonine	No	1.1202 ± 0.2821	0.6730
	Yes	1.1678 ± 0.2801	
Glutamine	No	0.6855 ± 0.2518	0.5414
	Yes	0.5633 ± 0.1466	
Succinic acid	No	0.3069 ± 0.2121	0.8884
	Yes	0.2595 ± 0.1406	
Glucose	No	1.2247 ± 0.6158	0.4807
	Yes	1.0736 ± 0.7355	
Tyrosine	No	0.0520 ± 0.0442	0.6058
	Yes	0.0552 ± 0.0599	
Phenylalanine	No	0.1449 ± 0.0771	0.8148
	Yes	0.1495 ± 0.0880	

Results of the Mann-Whitney *U* test. Metabolite differences between PP without neoadjuvant chemotherapy and PP with neoadjuvant chemotherapy (univariate analysis, nonparametric test)

**Table 4:** Impact of neoadjuvant chemotherapy on pancreatic adenocarcinoma (PA)

Metabolite	Neoadjuvant chemotherapy	Mean (nmol/mg)	*p* value
Ascorbate	No	0.3988 ± 0.2221	0.6648
	Yes	0.4062 ± 0.2227	
Aspartic acid	No	0.6229 ± 0.2255	0.0017
	Yes	0.9658 ± 0.6074	
Choline	No	0.8849 ± 0.3887	0.6600
	Yes	0.9539 ± 0.4702	
Creatine	No	1.3561 ± 0.5057	0.4285
	Yes	1.4464 ± 0.5971	
Ethanolamine	No	0.6148 ± 0.2620	0.1958
	Yes	0.7511 ± 0.4177	
Glutamate	No	2.4650 ± 0.7699	0.0908
	Yes	2.8070 ± 0.9767	
Glycerol	No	2.7550 ± 1.1994	0.1339
	Yes	3.5300 ± 2.6914	
Glycine	No	2.2663 ± 0.8577	0.1265
	Yes	2.9025 ± 1.8798	
Glycerophosphocholine	No	1.0319 ± 0.5029	0.7421
	Yes	1.0810 ± 0.7982	
Lactate	No	16.1370 ± 4.7272	0.7969
	Yes	15.9030 ± 5.3324	
Phosphorylcholine	No	0.9764 ± 0.5008	0.9092
	Yes	0.9518 ± 0.4822	
Taurine	No	4.4630 ± 1.4062	0.8886
	Yes	4.3490 ± 1.3425	
Threonine	No	1.1931 ± 0.4229	0.6273
	Yes	1.2957 ± 0.6961	
Glutamine	No	0.6394 ± 0.2272	0.5864
	Yes	0.6870 ± 0.2668	
Succinic acid	No	0.1762 ± 0.0763	0.4400
	Yes	0.1866 ± 0.0739	
Glucose	No	1.3141 ± 1.1020	0.0813
	Yes	2.3227 ± 2.7375	
Tyrosine	No	0.0730 ± 0.0493	0.3140
	Yes	0.1097 ± 0.1479	
Phenylalanine	No	0.1789 ± 0.0811	0.1622
	Yes	0.1097 ± 0.1958	

Results of the Mann-Whitney *U* test. Metabolite differences between PA without neoadjuvant chemotherapy and PA with neoadjuvant chemotherapy (univariate analysis, nonparametric test)

**Table 5:** Metabolite differences according to survival rate

Metabolite	No neoadjuvant chemotherapy	Mean (nmol/mg)	*p* value
Ascorbate	LongSurv	0.3988 ± 0.2221	0.5414
	ShortSurv	0.4062 ± 0.2227	
Aspartic acid	LongSurv	0.6229 ± 0.2255	0.7430
	ShortSurv	0.9658 ± 0.6074	
Choline	LongSurv	0.8849 ± 0.3887	0.0150
	ShortSurv	0.9539 ± 0.4702	
Creatine	LongSurv	1.3561 ± 0.5057	0.2766
	ShortSurv	1.4464 ± 0.5971	
Ethanolamine	LongSurv	0.6148 ± 0.2620	0.0078
	ShortSurv	0.7511 ± 0.4177	
Glutamate	LongSurv	2.4650 ± 0.7699	0.2766
	ShortSurv	2.8070 ± 0.9767	
Glycerol	LongSurv	2.7550 ± 1.1994	0.6730
	ShortSurv	3.5300 ± 2.6914	
Glycine	LongSurv	2.2663 ± 0.8577	0.2359
	ShortSurv	2.9025 ± 1.8798	
Glycerophosphocholine	LongSurv	1.0319 ± 0.5029	0.7430
	ShortSurv	1.0810 ± 0.7982	
Lactate	LongSurv	16.1370 ± 4.7272	0.0360
	ShortSurv	15.9030 ± 5.3324	
Phosphorylcholine	LongSurv	0.9764 ± 0.5008	0.6730
	ShortSurv	0.9518 ± 0.4822	
Taurine	LongSurv	4.4630 ± 1.4062	0.4807
	ShortSurv	4.3490 ± 1.3425	
Threonine	LongSurv	1.1931 ± 0.4229	0.3213
	ShortSurv	1.2957 ± 0.6961	
Glutamine	LongSurv	0.6394 ± 0.2272	0.1672
	ShortSurv	0.6870 ± 0.2668	
Succinic acid	LongSurv	0.1762 ± 0.0763	0.0747
	ShortSurv	0.1866 ± 0.0739	
Glucose	LongSurv	1.3141 ± 1.1020	0.4807
	ShortSurv	2.3227 ± 2.7375	
Tyrosine	LongSurv	0.0730 ± 0.0493	0.0592
	ShortSurv	0.1097 ± 0.1479	
Phenylalanine	LongSurv	0.1789 ± 0.0811	0.0055
	ShortSurv	0.1097 ± 0.1958	

Results of the Mann-Whitney *U* test (univariate analysis, nonparametric test). No neoadjuvant chemotherapy. *LongSurv* long-term survivors, *ShortSurv* short-term survivors

Continuous variables are expressed as mean ± standard deviation (SD). The Mann-Whitney *U* test was performed to compare the metabolites’ concentrations of (1) PP and PA (both without neoadjuvant chemotherapy), (2) PP without and PP with neoadjuvant chemotherapy, (3) PA without and PA with neoadjuvant chemotherapy, and (4) long-term and short-term survival in patients with PA (Tables 2, 3, 4, and 5). The Mann-Whitney U tests were performed using R software [30].

Receiver operating characteristic (ROC) curves and Kaplan-Meier curves were used to perform a survival analysis and therefore to evaluate the clinical utility of metabolite quantification in the long-term survival characterization (R software [30]).

## Results

All the spectra obtained from the 123 analyzed specimens were of high quality without any obvious evidence of tissue necrosis. A total of 31 metabolites were identified within the range of 7.50–0.70 ppm from the spectra obtained from all pancreatic tissue samples (Table 1). Among the 31 identified metabolites, only 18 metabolites were quantified: only well-defined peaks with no overlapping in the 1D CPMG spectra were selected for quantification.

**Fig. 1:**
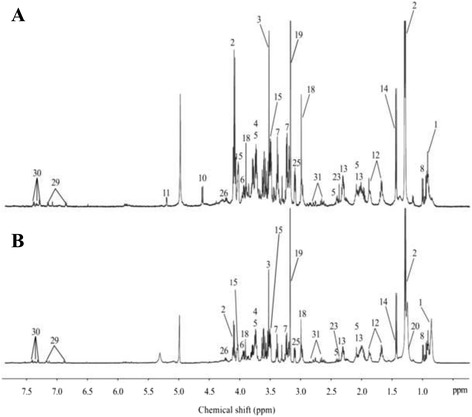
HRMAS NMR spectra of pancreatic healthy tissue (PP). **a** PP without neoadjuvant chemotherapy (*n* = 9), **b** PP with neoadjuvant chemotherapy (*n* = 8). The spectra metabolic contents are directly comparable because the intensity of each spectrum was normalized with respect to the weight of the analyzed sample. For display purposes, the amplitudes of the choline peak at 3.23 ppm, the glycine peak at 3.56 ppm, and the lactate peak at 1.33 ppm were graphically shortened. Metabolite assignments are given in Table 1

**Fig. 2:**
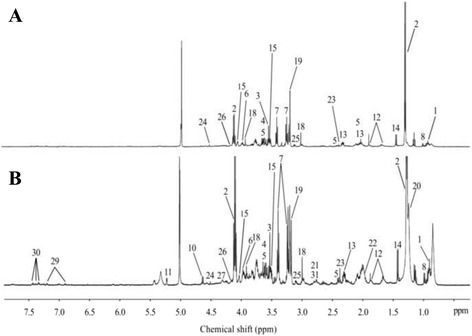
HRMAS NMR spectra of pancreatic adenocarcinoma (PA). **a** PA without neoadjuvant chemotherapy (*n* = 44), **b** PA with neoadjuvant chemotherapy (*n* = 62). The spectra metabolic contents are directly comparable because the intensity of each spectrum was normalized with respect to the weight of the analyzed sample. For display purposes, the amplitude of the lactate peak at 1.33 ppm was graphically shortened. Metabolite assignments are given in Table 1

**Fig. 3:**
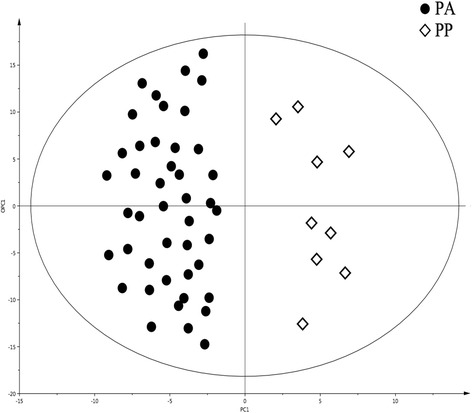
OPLS-DA comparing pancreatic adenocarcinoma (PA) with pancreatic healthy tissue (PP). A two-class model including 53 samples without neoadjuvant chemotherapy: 9 samples of PP and 44 of PA. A clear distinction between the different classes of tissues is shown in this model (R^2^Y = 0.79; Q^2^ = 0.62)

The representative 1D HRMAS NMR CPMG spectra of PP (healthy tissue) and PA samples (both without neoadjuvant chemotherapy) are shown in Figs. 1a and 2a. Some discriminant metabolites were highlighted using the Mann-Whitney *U* test. Choline (*p* = 0.0014), ethanolamine (*p* = 0.0226), glycerol (*p* = 0.0037), glycine (*p* = 0.0005), lactate (*p* = 0.0006), and taurine (*p* = 0.0021) were statistically significant between PP and PA (both without any neoadjuvant chemotherapy) (Table 2). Metabolomic profiles of PP and PA were clearly separated by a bi-component OPLS-DA (R^2^Y = 0.82; Q^2^ = 0.69) (Fig. 3). A higher concentration of myo-inositol and glycerol was shown in PP tissue samples. By contrast, a higher level of glucose, ascorbate, ethanolamine, lactate, and taurine was revealed in PA tissue samples.

### Impact of neoadjuvant chemotherapy on PP and PA

**Fig. 4:**
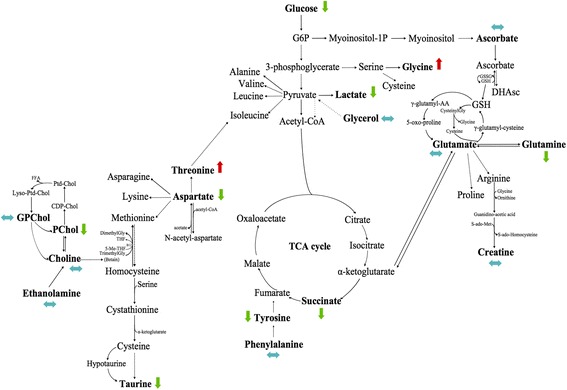
Impact of neoadjuvant chemotherapy on healthy tissue (PP). PP with neoadjuvant chemotherapy-related samples (*n* = 8) were compared to PP samples with no neoadjuvant chemotherapy (*n* = 9). Metabolic network analysis according to ADEMA results. The *red*, *green*, and *blue arrows*, respectively, indicate the metabolites that are predicted to increase, decrease, or remain stable in the population who received neoadjuvant chemotherapy

Seventeen samples from PP (healthy tissue) were included in this model: 8 samples received a neoadjuvant chemotherapy, while the 9 others did not (Fig. 1). No discriminant metabolites were found using the Mann-Whitney *U* test (Table 3). The network analysis showed that a decreased level of succinate, aspartate, taurine, phosphorylcholine, glucose, tyrosine, lactate, and glutamine was predicted in PP samples from patients with neoadjuvant chemotherapy. Moreover, a higher level of threonine and glycine was predicted in PP tissue samples from patients with neoadjuvant chemotherapy. The other metabolites were predicted to be equivalent between the two groups (Fig. 4).

**Fig. 5:**
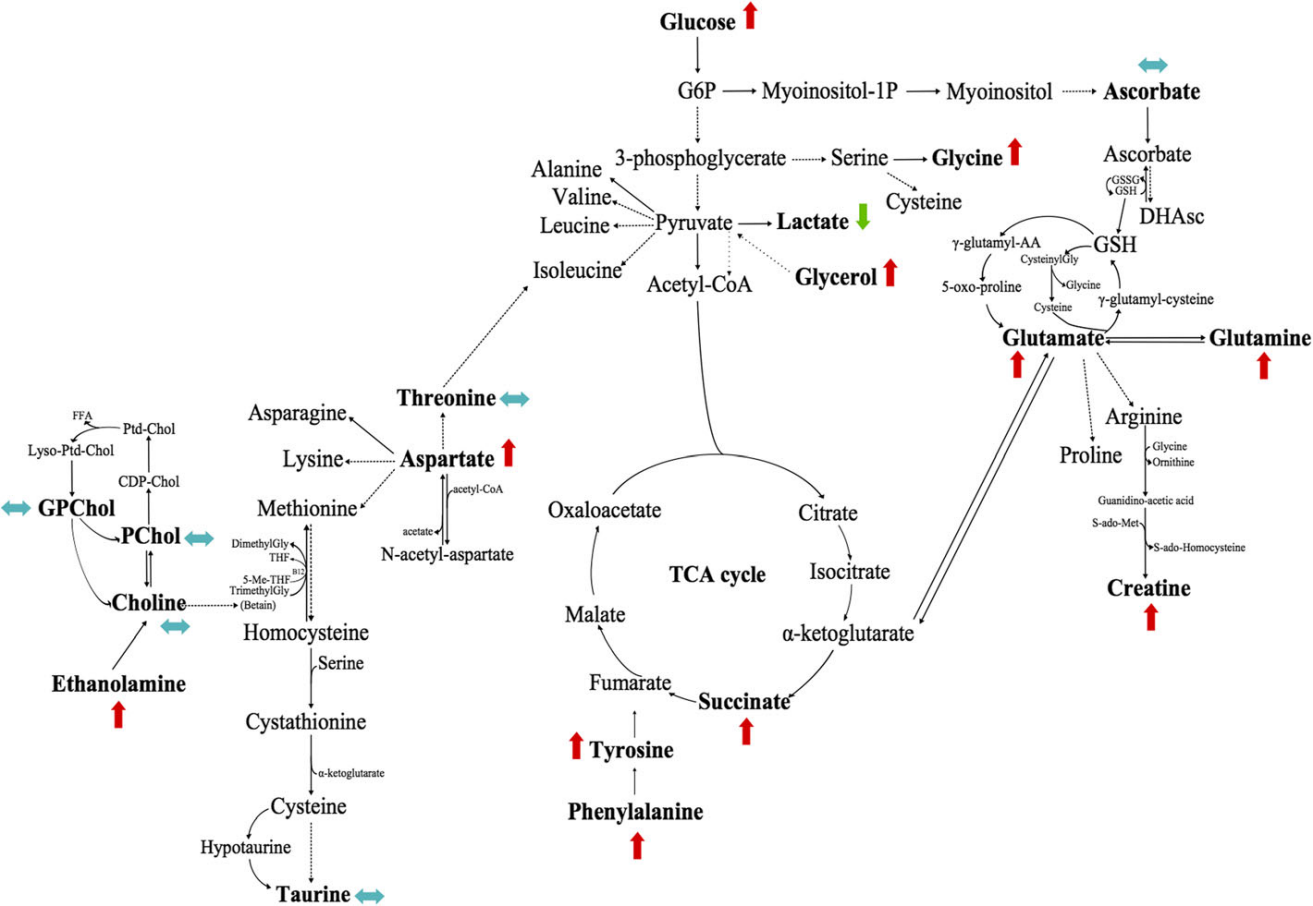
Impact of neoadjuvant chemotherapy on pancreatic adenocarcinoma (PA). PA with neoadjuvant chemotherapy-related samples (*n* = 62) were compared to PA without neoadjuvant chemotherapy (*n* = 44). Metabolic network analysis according to ADEMA results. The *red*, *green*, and *blue arrows*, respectively, indicate the metabolites that are predicted to increase, decrease, or remain stable in PA with neoadjuvant chemotherapy-related samples

Among the 106 samples of PA, 62 received neoadjuvant chemotherapy, while the 44 others did not (Fig. 2). Some discriminant metabolites were highlighted using the Mann-Whitney *U* test. Aspartate (*p* = 0.0017) was statistically significant between PA samples from patients who received neoadjuvant chemotherapy and those who did not (Table 4). As shown in Fig. 5, the network analysis revealed a decreased level of lactate, which was predicted in PA samples from patients with neoadjuvant chemotherapy. Moreover, higher levels of ethanolamine, tyrosine, phenylalanine, aspartate, glucose, glycerol, succinate, glycine, glutamate, glutamine, and creatine were predicted in PA tissue samples from patients with neoadjuvant chemotherapy. The other metabolites were predicted to be equivalent between the two groups.

For some of these analyses, the Mann-Whitney *U* test did not yield any significant differences, whereas ADEMA reports some changes for those metabolites. Thus, ADEMA appears more effective, as it compares different groups of metabolites, uses mutual information, and does not require a large population of samples.

### Long-term versus short-term survival in patients with PA

**Fig. 6:**
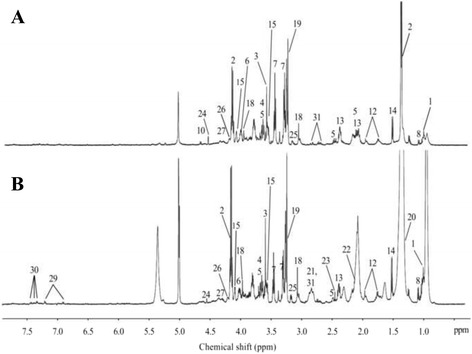
HRMAS NMR spectra of long-term and short-term survivors. **a** PA with long-term survival (*n* = 8), **b** PA with short-term survival (*n* = 9). The spectra metabolic contents are directly comparable because the intensity of each spectrum was normalized with respect to the weight of the analyzed sample. For display purposes, the amplitudes of the choline peak at 3.23 ppm, the fatty acids peak at 1.30 ppm, and the lactate peak at 1.33 ppm were graphically shortened. Metabolite assignments are given in Table 1

Patients’ characteristics are described in Table 6. The representative 1D HRMAS NMR CPMG spectra of long-term and short-term survivors are shown, respectively, in Fig. 6a and b. Seventeen samples were studied: 8 long-term survivors and 9 short-term survivors (both without any neoadjuvant chemotherapy). Some discriminant metabolites were highlighted using the Mann-Whitney *U* test. Choline (*p* = 0.0150), ethanolamine (*p* = 0.0078), lactate (*p* = 0.0360), and phenylalanine (*p* = 0.0055) were statistically significant between long-term and short-term survival in patients with PA (Table 5).

**Table 6:** Patients’ characteristics

	Age	Gender	Tumor size (mm)	CEA	CA 19-9	T	N	LNR	Margin (mm)	Differ	Vasc invas	G
LongS 1	70	F	50	8	52.3	3	1	1/57	2	W	No	IIB
LongS 2	70	M	25	2.1	202.56	3	0	0/21	1	M-P	No	IIA
LongS 3	63	M	35	2	200	4	0	0/16	3	M	Vein	III
LongS 4	66	F	50	1.1	1007	3	1	8/65	0	W-M	Vein	IIB
LongS 5	59	M	40	NA	NA	4	1	4/39	3	W-M	No	III
LongS 6	76	M	40	1.8	43.2	3	1	3/25	2	P	Vein	IIB
LongS 7	67	F	60	3.6	1626	3	1	4/43	3	M	No	IIB
LongS 8	69	F	25	1.4	220.4	3	0	0/16	3	M	No	IIA
ShortS 1	47	M	40	NA	112	3	1	6/64	2	P	No	IIB
ShortS 2	65	M	30	144	97.4	3	1	5/83	2	P	No	IIB
ShortS 3	78	F	30	3.6	250	3	1	NA	2	M	No	IIB
ShortS 4	72	F	35	2	13	3	1	NA	0	M-P	No	IIB
ShortS 5	82	F	25	NA	178	3	0	0/18	0	W	Vein	IIA
ShortS 6	49	F	30	2.3	451.5	3	1	6/42	1	P	Vein	IIB
ShortS 7	62	M	70	4.5	293.7	4	1	1/36	0	W	Vein	III
ShortS 8	60	M	40	4.2	916	3	1	18/33	0	Coll	Vein	IIB
ShortS 9	61	F	30	4.7	246.4	3	1	2/38	0	M	Vein	IIB

Seventeen samples from 17 patients with PA: 8 patients were classified as long-term survival patients (>3 years), while the 9 others had a short-term survival (<1 year). There was no significant difference in terms of T stage (*p* = 0.453), N+ status (*p* = 0.2), differentiation (*p* = 0.481), CA 19-9 (*p* = 0.236), or CEA (*p* = 0.322). There was a significantly larger resection margin in long-term survivors (2.13 mm vs 0.78 mm; *p* = 0.018). *LongS* long-term survival patients, *ShortS* short-term survival patients, *CEA* carcinoembryonic antigen, *NA* not available, *CA 19-9* carbohydrate antigen 19-9. *T* and *N* describing the tumor/node/metastasis: *T* tumor, *N* lymph nodes; *LNR* lymph node ratio, *Differ* differentiation, *W* well-differentiated, *P* poorly differentiated, *M* moderately differentiated, *Coll* colloid, *Vasc invas* vascular invasion, *G* grading

**Fig. 7:**
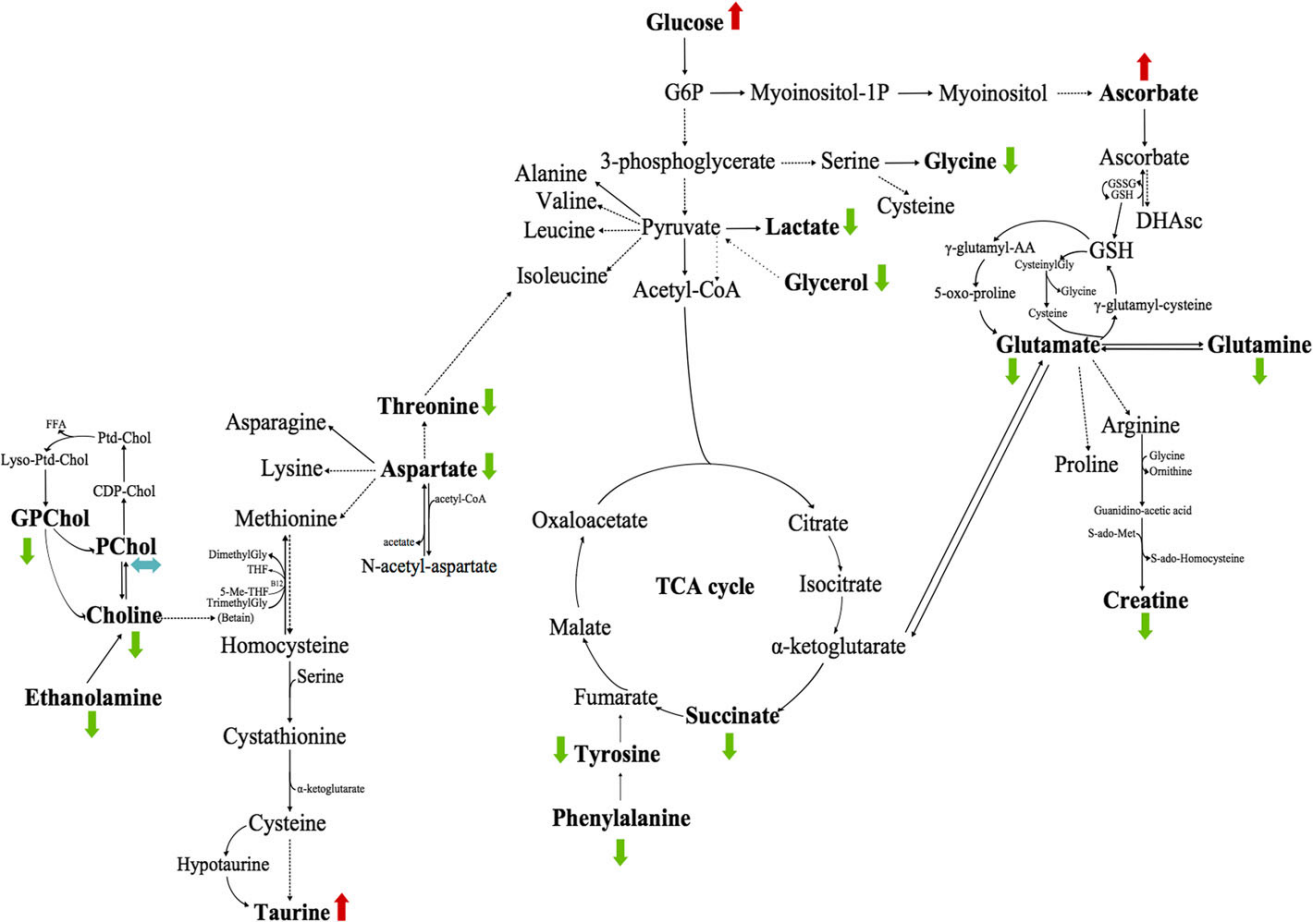
Metabolic network analysis enables pancreatic adenocarcinoma (PA) prognostication. Long-term survival-related samples (*n* = 8) were compared to short-term survival samples (*n* = 9) according to ADEMA results. No neoadjuvant chemotherapy was used. The *red*, *green*, and *blue arrows*, respectively, indicate the metabolites that are predicted to increase, decrease, or remain stable in long-term survivors

The network analysis showed higher levels of glucose, ascorbate, and taurine; this was predicted in long-term survivors. Moreover, decreased levels of choline, ethanolamine, glycerophosphocholine, phenylalanine, tyrosine, aspartate, threonine, succinate, glycerol, lactate, glycine, glutamate, glutamine, and creatine were predicted in long-term survivors. Phosphorylcholine was predicted to be equivalent between the two groups (Fig. 7).

### Survival analysis

**Fig. 8:**
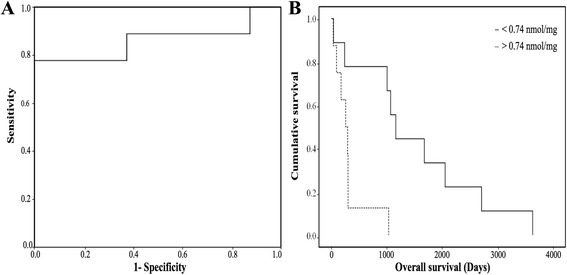
Ethanolamine concentration as a single metabolic biomarker predicting the overall survival in patients with PA. **a** ROC and **b** Kaplan-Meier curves obtained from the analysis of ethanolamine concentrations for the diagnosis of long-term survival in patients with PA. The AUC was 0.861 ± 0.101, the threshold value was 0.740 nmol/mg, and sensitivity and specificity were, respectively, equal to 77.80% and 75.00%. The Kaplan-Meier curve shows differences between long-term and short-term survival patients. The *p* value was 0.005 (for the log-rank test)

Statistical significant differences were highlighted when we focused on the comparison between long-term and short-term survival patients. Only the use of ethanolamine as a single screening test showed a higher accuracy in distinguishing long-term from short-term survivors. As shown in Fig. 8, samples with a decreased level of ethanolamine had a high probability of being assigned to long-term survivors. All the patients showing a low level of ethanolamine were long-term survival patients. The area under the curve (AUC) was 0.861 ± 0.101. The optimal ethanolamine threshold was 0.740 nmol/mg when distinguishing long-term from short-term survivors. With this threshold point, sensitivity and specificity were, respectively, 77.80% and 75.00% (Fig. 8a). The predictive positive value was 47.00% and the negative predictive value was 52.90%. A Kaplan-Meier analysis showed that the overall survival probabilities were significantly higher in patients with low tumor ethanolamine concentrations compared to those with high tumor ethanolamine concentrations (Fig. 8b).

## Discussion

To the best of our knowledge, this is the first study that has evaluated the metabolome of intact tissues for PP and PA.

Preoperative assessment of resectability of PA is not yet an adequate way of predicting survival. However, given the morbidity and mortality of CDP, a better evaluation of the balance between risks and benefits is a complex quest. Indeed, serum markers such as carcinoembryonic antigen (CEA) or carbohydrate antigen (CA 19-9) are poorly correlated to long-term results and should not be used for contraindicating the only potentially curative treatment of this often fatal disease. Up to now only histological parameters have enabled surgeons to retrospectively evaluate the potential benefit of CDP, but these parameters are available only after surgery. Moreover, none of these parameters is really specific for predicting the necessity of pancreatic resection. In order to evaluate the benefit of pancreatic resection, long-surviving patients were compared to short-term survivors. Of note, among the long-term survivors, two of them showed an infra-millimetric margin, and three had histological invasion of the portal vein (Table 6). Thus, the use of currently described predictive factors should not lead to contraindicating resection in patients who can otherwise tolerate surgery [31]. The use of a more specific marker tightly linked to the pathophysiology of pancreatic cancer would be of particular interest.

HRMAS NMR provides new insights into the relationships between metabolic pathways and pancreatic cancers. This method allows for the identification of cell membranes and phospholipid metabolism, cellular energy production via neoglucogenesis, the tricarboxylic acid (TCA) cycle, and oxidative stress. The majority of PAs have KRAS mutation (90% with activating mutations in this oncogene) [32–34].

Our results about PP’s metabolome show a higher level of lactate, glucose, phosphorylcholine, taurine, aspartate, lactate, glutamine, and succinate in patients who received neoadjuvant chemotherapy (gemcitabine). Mutations in TCA cycle enzymes are known to promote cancer development and growth. For example, mutations in succinate dehydrogenase (SDH), fumarate hydratase (FH), and isocitrate dehydrogenase 1 and 2 (IDH1, IDH2) can be cited [35, 36]. Moreover, these mutations have been associated with distinct cancer subsets and different patient prognoses [37, 38]. Mutations in TCA cycle enzymes have also been associated with measurable changes in the levels of metabolites. Nevertheless, these mutations have not been a major feature of PA. Reprogrammed cellular metabolism has increasingly become an obvious field of research for PA. Research has also focused on the alterations that involve the TCA cycle and mutant KRAS-induced tumor cell dependencies for glucose, glutamine, and extracellular protein.

Our results about PA’s metabolome show a higher level of lactate in patients who did not receive any neoadjuvant chemotherapy. Elevated expression of lactate dehydrogenase A (LDHA) is highlighted in breast and colorectal cancers [39, 40] and has been recently identified in pancreatic cancers [41]. Some studies have already shown the different steps where LDHA is involved (tumor initiation, maintenance, and progression) [42, 43]. Given the elevated glycolysis in PA, leading to increased lactate production, the ability to utilize lactate could provide an additional advantage to PA cells [1]. Indeed, the inhibition of glycolysis through suppressing LDHA expression by RNA interference decreased the growth of PA cells [44]. Our study may provide new targets to treat PA. When patients who received neoadjuvant chemotherapy and patients who did not were compared, neoadjuvant chemotherapy seemed to have an inhibiting effect on LDHA expression. A higher level of lactate in patients who did not receive any neoadjuvant chemotherapy showed that survival of PA was highly dependent on LDHA activity in a hypoxic environment. Then, this higher level of lactate could be linked to improved responses to neoadjuvant chemotherapy. Indeed, lactate might be a predictive marker for assessing the response of tumor cells to neoadjuvant chemotherapy. Finally, lactate could be correlated with tumor neoadjuvant chemotherapy in predicting responses to this therapy. Elevated levels of lactate are prognostic biomarkers for poor survival in several cancers. Our findings suggest that lactate could be an important marker for screening the efficiency of cancer treatments.

A decreased level of glucose has also been shown in PA without neoadjuvant chemotherapy. As described in the work of Koong et al. [45] and Guillaumont et al. [46], areas within PA tumors are hypoxic, and this has implications for the cellular metabolism. Hypoxia promotes tumor growth by particularly stimulating glycolysis.

When PP and PA, both without chemotherapy, were compared, some discriminant metabolites were highlighted, particularly lactate and taurine. Our study shows a higher level of lactate and taurine in patients with PA. These results are consistent with the work of Wang et al. [47]. The work of Nishiumi et al. has also underlined elevated levels of lactate in the patients’ serum [48]. As described in the work of Wang et al., higher levels of taurine have also been reported in the literature, for several types of cancer. As previously explained, this increased lactate level in PA could come from hypoxia. Our study is in accordance with other studies that have found higher levels of taurine in different types of cancer, probably due to apoptosis [49, 50]. Even so, further studies will be needed to understand the complex biology of that particular type of cancer. If confirmed by other studies, this could deepen our knowledge of pancreatic cancer pathogenesis and might also lead to the identification of new targets for diagnosis, early detection, imaging, or even future therapeutic options.

Our results about PA’s metabolome in short-term survivors are in accordance with other studies that found a higher level of choline in several cancers. The role of choline kinase α (Chk-α) in malignant transformation and progression in several cancers has been well described in the literature. Increased levels and activity of Chk-α have been observed in human breast [51], colorectal [52], lung [52, 53], prostate [52], ovarian [54], and more recently in endometrial [55] and pancreatic cancers [56].

The altered choline metabolism we observed is possibly due to an overexpression of Chk-α. Increased levels of choline could be due to an increase in the membrane activity, particularly due to enhanced cellular proliferation. This activity is increased in inflammatory or tumoral conditions. In the most malignant cases, this membrane activity is much higher still. Increased levels of choline are also due to malignant transformation, and particularly due to an overexpression of Chk-α. Hypoxia may also affect choline phosphorylation through effects on Chk-α and transporters [57]; in turn, choline phosphorylation may be affected by hypoxia through effects on Chk-α and transporters. In some studies, particularly in prostate cancer [58], increased levels in choline have been described, leading to the development of ^18^F-fluorocholine positron emission tomography (PET) imaging in order to detect the increased uptake and the phosphorylation of the tracer. Consequently, our results are in agreement with observations in the literature and could represent the biological substrate and justification to the use of ^18^F-fluorocholine PET imaging in pancreatic cancers [59–61]. Metabolic targets in choline phospholipid metabolism may provide new therapeutic options for PAs that have severely limited options. Moreover, a decreased amount of succinate was shown by the network analysis in long-term survivors compared to short-term survivors, suggesting a decreased activity of the TCA cycle. PAs from long-term survivors also show decreased levels of threonine, aspartate, glycerol, glutamate, and glutamine compared to those from short-term survivors. Glutamine has been particularly studied for its role in cancer metabolism because it appears to be required for the growth of many types of tumors [62]. Decreased levels of creatine and lactate were shown in long-term survival patients too. Other studies showed the relationship between lower levels of creatine, lactate, and choline and overall survival [63]. Understanding the different metabolic links within pancreatic cancer is a promising approach to identifying novel prognostic markers (long-term survival) and therapeutic programs in patient care [1].

Short-term survivors showed higher levels of choline, glycerophosphocholine, ethanolamine, and fatty acids, as depicted in Fig. 6b (respectively, numbers 19, 25 and 21, 22). Fatty acids are a major factor in the growth of tumor cells. Some explorations have begun in order to study the PA metabolism and more precisely the role of individual complex fatty acids. Indeed, as detailed in the work of Guo et al. [64] and Zadra et al. [65], fatty acids can be both pro-tumorigenic and anti-tumorigenic, as described for various cancers, making their biology difficult to explain. Reducing the levels of certain fatty acids seems to be important for PA. But, we have to determine first which fatty acids are cytotoxic for tumor cells and which fatty acids provide the tumor with metabolic substrates [1]. Focusing on ethanolamine, the survival analysis showed that survival was longer for patients with lower tumoral ethanolamine concentrations. The assessment of ethanolamine concentration can be clinically relevant as a single metabolic biomarker for distinguishing long-term survivors from short-term survivors in patients with PA.

The current study demonstrates that metabolomics profiling may provide prognostic information in patients with pancreatic cancer. Research has usually focused on enzymatic steps within the TCA cycle, as it potentially influences the progression of disease, as well as on alterations of the phospholipid metabolism within the choline/ethanolamine membrane.

Only very few patients are needed to build a model that can predict oncological outcome in pancreatic cancer very accurately; this fact alone should help in promoting our technique.

We acknowledge some limitations to the present study. First, the number of patients is limited, particularly regarding the long-term survivors included into our study. Pancreatic cancers are extremely aggressive and have one of the poorest prognoses among all cancers. Thus, very few patients get a chance of long-term survival, and even less so without any neoadjuvant chemotherapy. Second, further studies should take into account other elements that affect the survival of patients. We believe these data are preliminary and should be validated in further series. We encourage others to validate the findings and to carry out multicenter studies. Indeed, in order to include co-factors, the patient population needs to be significantly enhanced (because PAs have one of the poorest prognoses among all cancers). Third, we acknowledge that a comparative effectiveness study should be performed in real time in order to evaluate usual serum markers versus HRMAS NMR spectroscopy before making any definitive conclusions. Fourth, due to the limited number of patients and the preliminary character of our results, it is difficult to draw a conclusion about the level of ethanolamine. For the moment, there is no specific concept. Fifth, although we acknowledge that the assessment of ethanolamine concentration can be clinically relevant as a single metabolic biomarker for distinguishing long-term survivors from short-term survivors in patients with PA, this result should be put into perspective. Indeed, sensitivity and specificity are respectively 77.80% and 75%. Lastly, this study was retrospective and may involve some bias that could have been unaccounted for.

Metabolomics analysis could be validated as an intraoperative discriminant method for distinguishing healthy tissues from PA tissues. This could deepen our knowledge of PA metabolism and may also lead to the identification of new targets for diagnosis, imaging, or future therapeutic options. If these results are confirmed in further studies, it is expected that the role of intraoperative HRMAS NMR spectroscopy could then be evaluated in the setting of PA. This approach, which we call “metabolomics-guided surgery,” could help surgeons to extend the excision if necessary. Since HRMAS NMR spectroscopy enables rapid characterization of intact tissue, it could also be used as an intraoperative method. HRMAS analysis only takes 20 min. Data analysis is also very quick (<10 min). Furthermore, the cost is < $50 per sample.

## Conclusions

In conclusion, as we are able to distinguish PP from PA, we could imagine using this technique to analyze several samples collected from the excision cavity in addition to tissue tumor samples. This last step could help surgeons in the detection of residual tumor cells in the excision cavity and the control of margins. This approach could be used in clinical routine for prediction of long-term survival in patients with PA; indeed, this information can be obtained in 20 min during surgery. Finally, due to the high morbidity and mortality during surgery, we could also imagine using this technique before surgery, with the use of endoscopic or percutaneous biopsy, although these remain invasive techniques. On the whole, our own technique could prove useful and have a positive impact on patient care.

## Additional file

Additional file 1: Values of metabolites'quantification. (XLSX 80 kb)

## Abbreviations

CDP: Cephalic duodeno-pancreatectomy
CPMG: Carr-Purcell-Meiboom-Gill
HRMAS: High-resolution magic angle spinning
NMR: Nuclear magnetic resonance
OPLS-DA: Orthogonal partial least square-discriminant analysis
PA: Pancreatic adenocarcinoma
PCA: Principal component analysis
PP: Pancreatic parenchyma
